# The global distribution and diversity of protein vaccine candidate antigens in the highly virulent *Streptococcus pnuemoniae* serotype 1

**DOI:** 10.1016/j.vaccine.2016.12.037

**Published:** 2017-02-07

**Authors:** Jennifer E. Cornick, Özlem Tastan Bishop, Feyruz Yalcin, Anmol M. Kiran, Benjamin Kumwenda, Chrispin Chaguza, Shanil Govindpershad, Sani Ousmane, Madikay Senghore, Mignon du Plessis, Gerd Pluschke, Chinelo Ebruke, Lesley McGee, Beutel Sigaùque, Jean-Marc Collard, Stephen D. Bentley, Aras Kadioglu, Martin Antonio, Anne von Gottberg, Neil French, Keith P. Klugman, Robert S. Heyderman, Mark Alderson, Dean B. Everett, for the PAGe consortium

**Affiliations:** a Malawi-Liverpool Wellcome Trust Clinical Research Programme, Queen Elizabeth Central Hospital, Blantyre, Malawi; b Clinical Infection, Microbiology and Immunology, Institute of Infection and Global Health, University of Liverpool, Liverpool L69 7BE, UK; c Research Unit in Bioinformatics (RUBi), Department of Biochemistry and Microbiology, Rhodes University, Grahamstown, South Africa; d Pathogen Genomics, Wellcome Trust Sanger Institute, Wellcome Trust Genome Campus, Hinxton, Cambridge CB10 1SA, UK; f Centre de Recherche Médicale et Sanitaire, Niamey, Niger; g Medical Research Council, Banjul, Gambia; h Division of Translational and Systems Medicine, Microbiology and Infection Unit, The University of Warwick, UK; i Centre for Respiratory Diseases and Meningitis, National Institute for Communicable Diseases, A Division of the National Health Laboratory Service, Johannesburg, South Africa; j School of Pathology, Faculty of Health Sciences, University of the Witwatersrand, Johannesburg, South Africa; k Swiss Tropical and Public Health Institute, Basel, Switzerland; l Centers for Disease Control and Prevention, Atlanta, USA; m Centro de Investigação em Saúde da Manhiça, Maputo, Mozambique; n Faculty of Infectious and Tropical Diseases, London School of Hygiene and Tropical Medicine, London, UK; o Hubert Department of Global Health, Rollins School of Public Health, Emory University, USA; p PATH, Seattle, WA, USA

**Keywords:** Protein modelling, Structural diversity, Antigenic diversity, Antigenic profiling, Variant, Pneumococcal disease, Multi-valent, PCV

## Abstract

Serotype 1 is one of the most common causes of pneumococcal disease worldwide. Pneumococcal protein vaccines are currently being developed as an alternate intervention strategy to pneumococcal conjugate vaccines. Pre-requisites for an efficacious pneumococcal protein vaccine are universal presence and minimal variation of the target antigen in the pneumococcal population, and the capability to induce a robust human immune response. We used *in silico* analysis to assess the prevalence of seven protein vaccine candidates (CbpA, PcpA, PhtD, PspA, SP0148, SP1912, SP2108) among 445 serotype 1 pneumococci from 26 different countries, across four continents. CbpA (76%), PspA (68%), PhtD (28%), PcpA (11%) were not universally encoded in the study population, and would not provide full coverage against serotype 1. PcpA was widely present in the European (82%), but not in the African (2%) population. A multi-valent vaccine incorporating CbpA, PcpA, PhtD and PspA was predicted to provide coverage against 86% of the global population. SP0148, SP1912 and SP2108 were universally encoded and we further assessed their predicted amino acid, antigenic and structural variation. Multiple allelic variants of these proteins were identified, different allelic variants dominated in different continents; the observed variation was predicted to impact the antigenicity and structure of two SP0148 variants, one SP1912 variant and four SP2108 variants, however these variants were each only present in a small fraction of the global population (<2%). The vast majority of the observed variation was predicted to have no impact on the efficaciousness of a protein vaccine incorporating a single variant of SP0148, SP1912 and/or SP2108 from *S. pneumoniae* TIGR4. Our findings emphasise the importance of taking geographic differences into account when designing global vaccine interventions and support the continued development of SP0148, SP1912 and SP2108 as protein vaccine candidates against this important pneumococcal serotype.

## 1 Introduction

*Streptococcus pneumoniae* is a human nasopharyngeal commensal that can invade normally sterile sites to cause invasive pneumococcal disease (IPD), including bacteraemia and meningitis [1]. Annually, IPD accounts for more than 800,000 deaths in children under five years old, mainly in the developing world [2], [3].

There are >90 pneumococcal serotypes, each of which produce a biochemically distinct capsular polysaccharide (CPS) and vary in propensity to cause invasive disease [4]. Serotype 1 is one of the most common causes of IPD worldwide. In Africa, it is responsible for 11.7% of all IPD cases [5]. In contrast to other serotypes, serotype 1 is associated with outbreaks in closed communities [6] and lethal meningitis outbreaks in West Africa [7], [8]. The high burden of serotype 1 IPD emphasizes the need for an effective vaccine against this serotype. The 10- and 13-valent pneumococcal conjugate vaccines (PCV10 and PCV13) have been rolled out across Africa with support from the GAVI alliance (www.gavi.org), both formulations incorporate serotype 1, however the full impact of these vaccines across the continent is not yet known [9].

A major limitation of PCVs is they only elicit protective antibodies against the serotypes included in the vaccine formulation. As a result non-vaccine serotypes can increase in frequency in IPD and carriage post PCV introduction, as observed following PCV7 introduction in the USA [10]. Furthermore, vaccine serotypes can alter/exchange portions of their CPS locus to escape host antibodies, a phenomenon known as ‘capsule switching’ [11]. The ability of pneumococci to continuously alter their genotype and thereby evade vaccine selective pressure is often referred to as ‘Red Queen’ dynamics; it has been suggested that Red Queen dynamics must be taken into account when designing new vaccine strategies [12]. Alternative serotype-independent protein vaccines, which avoid Red Queen dynamics by targeting widely distributed pneumococcal proteins are therefore in development based on conserved, [13]. PATH (www.path.org) is committed to develop protein-based vaccines tailored for the developing world and has highlighted seven promising candidates: choline binding protein (CbpA), pneumococcal-binding protein A (PcpA), pneumococcal histidine triad protein (PhtD), pneumococcal surface protein C (PspC), SP0148 a putative ABC transporter protein, SP1912 a putative thioredoxin and SP2108 a putative sugar ABC transporter substrate binding protein. CbpA has been shown to mediate antibody protection against pneumococcal pneumonia, as has PspC [14]. PcpA and PhtD exhibited promising immunogenicity and safety profiles in monovalent and bivalent formulations in phase 1 clinical trials [15]. A trivalent formulation of SP0148, SP1912, and SP2108 was reported to be safe and immunogenic and to elicit strong T helper 17 cells (T_H_17) responses in healthy volunteers in phase 1 clinical trials [16], [17].

In addition to being immunogenic, a requisite for a protein vaccine candidate is that the selected antigen(s) is widely distributed in the target pneumococcal population. Variation in the amino acid sequence of the protein has the potential to reduce the immunogenicity of a vaccine based on only a single variant. Hence an additional requisite is minimal genomic diversity within the selected protein among clinical isolates. Here we consider seven protein vaccine candidates currently under investigation by PATH in a global collection of serotype 1 pneumococci, with a specific focus on African isolates, in order to assess their suitability as future vaccine candidates against this important serotype. We describe their prevalence’s and further investigate the sequence-based prediction of antigenic and structural diversity of three of them.

## 2 Methods

### 2.1 Isolate collection

The collection was previously subjected to Illumina sequencing and genome assembly by the Pneumococcal African Genomics Consortium (http://www.pagegenomes.org) (Supplementary Table S1); a detailed sampling description can be found elsewhere [18]. Isolates were recovered between 1994 and 2009 prior to the widespread introduction of PCV in Africa and were collected to encompass diversity with respect to geographic location, isolation, clinical source (carriage and disease) and patient age.

### 2.2 Protein identification

Digital primers were designed to ‘bind’ to the flanking 31 nucleotides from 5′ and 3′ ends of each vaccine candidate gene (Table 1), *in silico* PCR was performed using Perl scripts to search each gene primer pair against the assembled genomes and allowed for sequence variation in the ‘binding sites’. Nucleotide sequence between each primer pair was translated into amino acid sequence.

### 2.3 Variant determination

Amino acid sequences for SP0148, SP1912 and SP2108 were independently aligned, to identify different variants at the protein level. Each alignment was compared to the reference sequence SP0148 TIGR4 (Accession: NP_344690), SP1912 TIGR4 (Accession: ABJ54543) or SP2108 TIGR4 (Accession: ABJ55468); TIGR4 genes coding for these proteins where initially cloned in *Escherichia coli* for immunogenicity studies and phase I trials [17]. Each variant was assigned a unique numerical designation.

### 2.4 Antigenicity plots

Antigenicity patterns of SP0148, SP1912 and SP2108 were measured using the Hopps and Woods hydrophilicity scale (http://web.expasy.org/protscale/) with a window size of 9, which assigns a numerical hydrophilicity value to each amino acid and takes a moving average along the peptide chain. The point of highest local average hydrophilicity is consistently located in, or immediately adjacent to an antigenic determinant [19]. The hydrophilicity values were used to generate antigenicity plots for each variant. Antigenicity plots with different amplitudes/number of peak hydrophilicity points from the TIGR4 reference were considered to be ‘antigenically different’.

### 2.5 Prediction of functional effects of variations

PROVEAN [20], PhD-SNP (http://snps.biofold.org/phd-snp/) and PolyPhen-2 [21] were used to predict the impact of amino acid substitutions. PROVEAN includes bacterial protein analysis; PhD-SNP and PolyPhen-2 were developed to analyse human proteins; thus only the PROVEAN results alone or the consensus result of three programs was considered (Supplementary Table S2).

### 2.6 Homology modelling

TIGR4 reference sequences of SP0148, SP1912 and SP2108 were used as queries in HHpred search for template identification [22]. For SP0148, protein structure 4EQ9; for SP1912 2M70; for SP2108 2XD3 was identified. Modelling was performed by MODELLER v9.16 using very slow refinement option. 100 models were generated per case, and best models were selected according to DOPE Z scores.

## 3 Results

### 3.1 Distribution of protein vaccine candidates

**Fig. 1:**
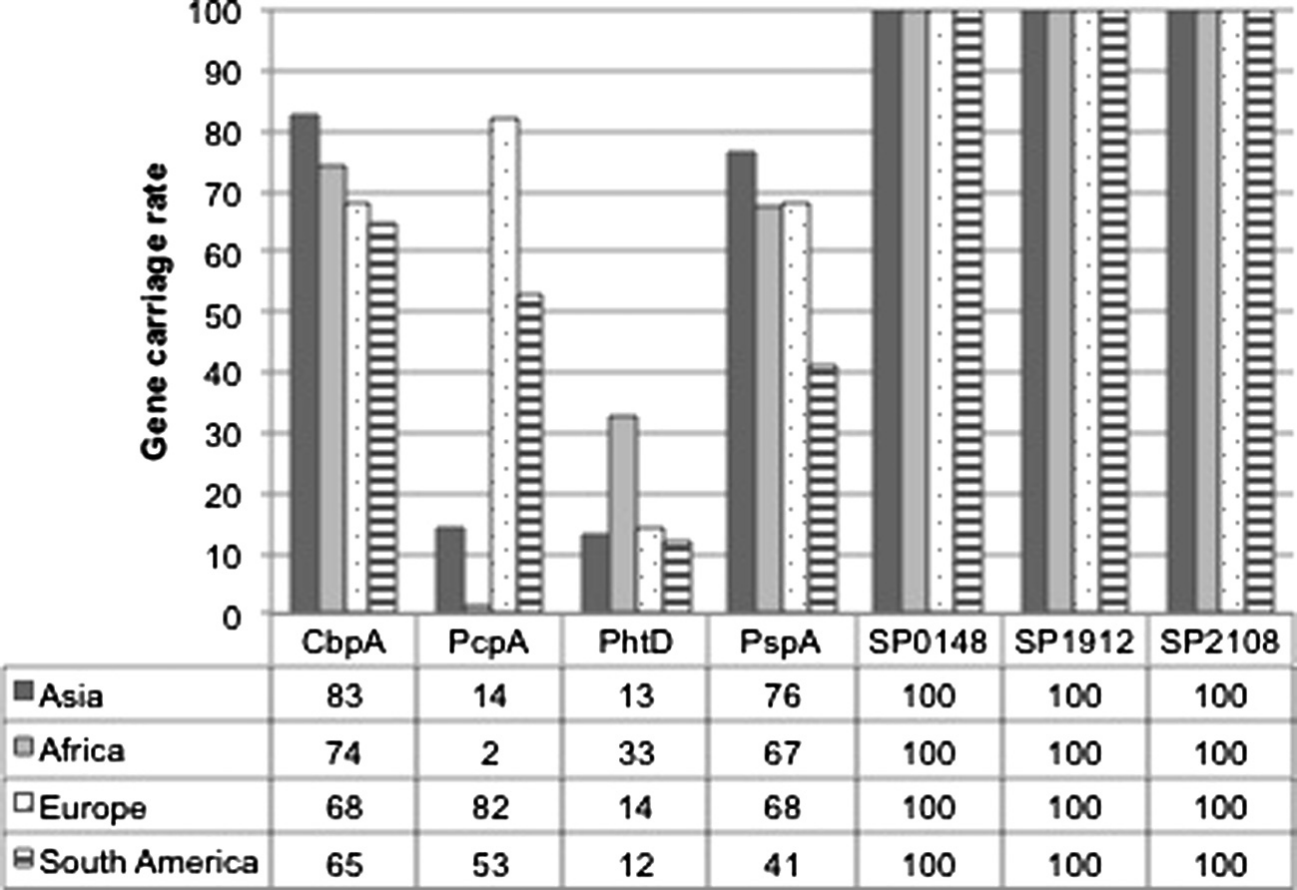
Global percentage distribution of seven *S. pneumoniae* protein vaccine candidates among serotype 1 pneumococci recovered from Asia (n = 76), Africa (n = 324), Europe (n = 28) and South America (n = 17), expressed as a percentage of the number of samples submitted from each continent.

An effective protein vaccine candidate needs to be based on a protein antigen that is widely distributed within the population. We therefore assessed the prevalence of seven-protein vaccine candidates within a global serotype 1 collection (n = 445) (Fig. 1). The African collection included 324 isolates, the non-African collection included 121 isolates spanning three continents (Asia = 76, Europe = 28, South America = 17). SP0148, SP1912 and SP2108 were identified in 100% (445/445), CbpA was identified in 76% (340/445), PspA in 68% (305/445), PhtD in 28% (125/445) and PcpA in 11% (49/445) of the study population. A monovalent protein vaccine based on CbpA, PcpA, PhtD or PspA would only provide partial coverage against serotype 1 globally. PspA was identified in 76% (58/76) of Asian isolates but only 41% (7/17) of South American isolates. Likewise, PcpA was identified in 82% (23/28) of the European isolates, but only 2% (6/324) and 14% (11/76) of the African and Asian isolates respectively.

### 3.2 Multi-valent vaccine coverage

Vaccines based on a combination of two or more proteins have been proposed to increase protein vaccine coverage. Table 2 shows the predicted coverage of combinations of CbpA, PcpA, PhtD and/or PspA against serotype 1 pneumococci. A combination of all four proteins would provide the highest coverage (86%) against serotype 1 pneumococci globally, only 2% higher than the predicated coverage from a tri-valent combination CbpA/PcpA/PspA (82%). Of the bi-valent combinations, CbpA/PcpA (79%) would provide the highest and PcpA/PhtD (37%) the lowest overall predicted global coverage.

### 3.3 Diversity of vaccine candidates

**Fig. 2:**
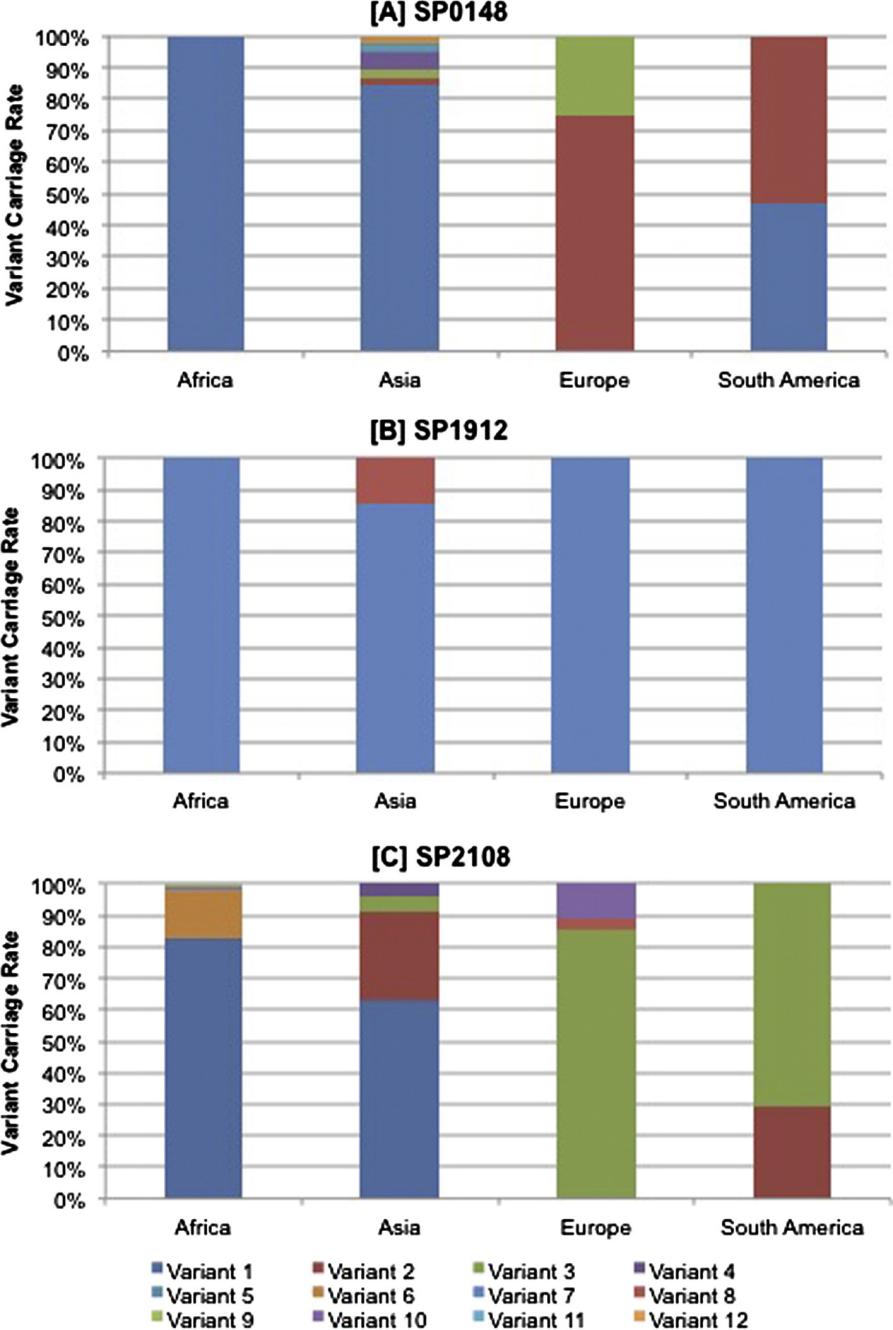
Global percentage distribution of amino acid variants of three *S. pneumoniae* protein vaccine candidates; [A] SP0148, [B] SP1912 and [C] SP2108. These three protein candidates were present in all serotype 1 pneumococci within the global serotype 1 study population.

The diversity of the protein vaccine candidates that showed 100% distribution, SP0148, SP1912 and SP2108, was investigated (Fig. 2, Table 3). Based on amino acid sequence variation, six variants of SP0148 were identified, all 276 amino acids in length. SP0148 variant-1 was the most dominant, present in 100% (324/324), 84% (64/76) and 47% (8/17) of the African, Asian and South American isolates respectively. Variant-1 was not identified in the European population; SP0148 variant-2 and -3 were present in 75% (21/28) and 25% (7/28) of the European population, respectively. In contrast to the dominance of a single variant in the African serotype 1 population, SP0148 showed a high degree of heterogeneity in the Asian serotype 1 isolates; all 6 variants were identified in the Asian population. The SP0148 variants shared between 91.3% (variant-3) and 99.3% amino acid (variant-2) identity with SP0148 TIGR4.

Only two variants of SP1912 were identified, both 99 amino acids in length. With the exception of a subset of the Asian isolates, all study isolates possessed an SP1912 variant-1 with 100% amino acid identify to SP1912 TIGR4. Variant-2 was identified in 15% (11/76) of the Asian isolates, 2% (11/445) of the overall study population showed 97% identity to SP1912 TIGR4.

Twelve variants of SP2108 were identified, all 426 amino acids in length. Variant-1 exhibited 100% identity to SP2108 TIGR4. All of the remaining variants exhibited above 99.3% identity to SP2108 TIGR4. SP2108 variant-1 was the most widely distributed variant, present in the African (82%. 272/332) and Asian (63%, 48/76) populations but not present in the European or South American isolates. Variant-3 was identified in the European (86%, 24/28) and South American (71%, 12/17) populations but not in any of the African or Asian.

### 3.4 Functional effects of variation

**Fig. 3:**
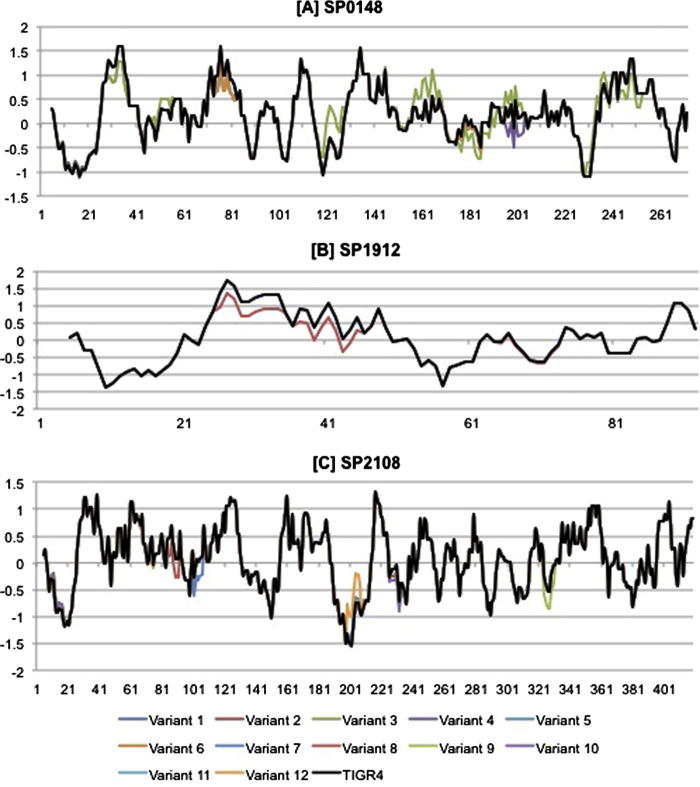
Comparison of the hydrophobicity plots of amino acid variants of three *S. pneumoniae* protein vaccine candidates [A] SP0148, [B] SP1912 and [C] SP2108**.** The vertical axis represents hydrophilicity values; the y axis indicates amino acid number. The region of maximal hydrophilicity in each plot is likely to be the antigenic site. The black data line represents the antigenicity values for each of the three proteins from the TIGR4 reference.

**Fig. 4:**
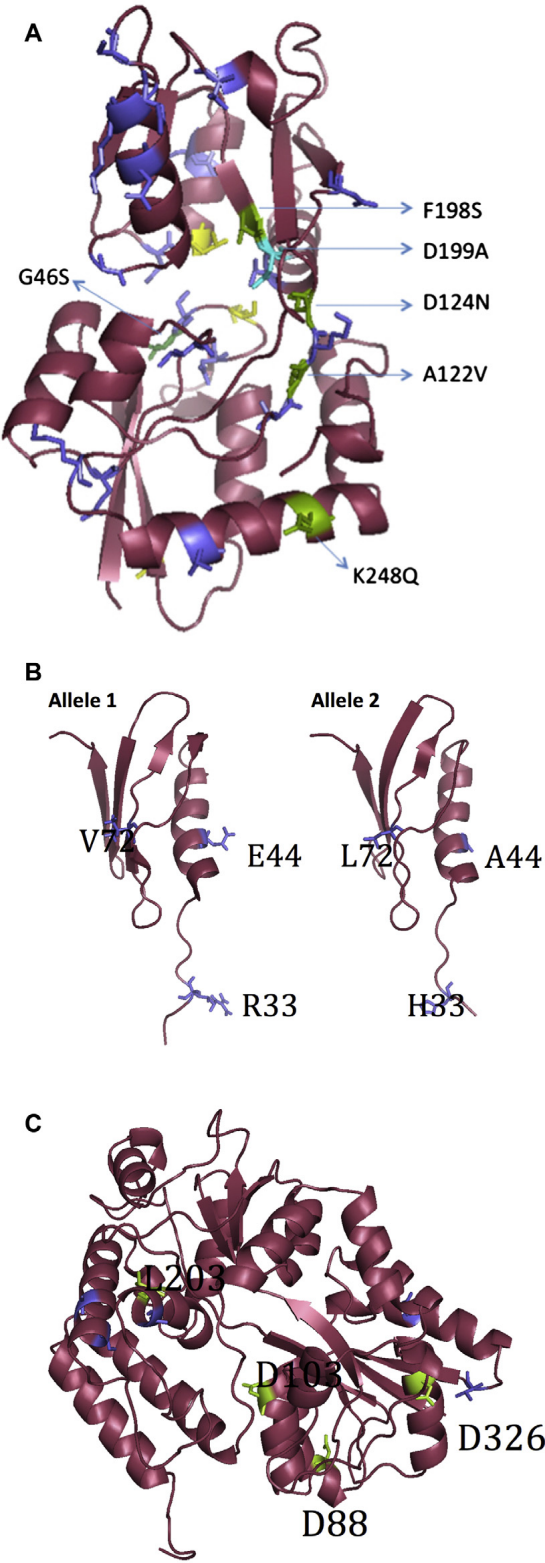
Cartoon representations of the protein structure of [A] SP0148 [B] SP1912 and [C] SP2108. [A] SP0148 – The locations of the variant-3 amino acid substitutions are mapped into the structure; green for the potential deleterious variations detected by PROVEAN only (also indicated by their residue numbers); purple for the non-deleterious variations. Additionally, the position of D199A variation, detected in variant 5 and agreed as deleterious by 3 SNP programs, is colored in turquoise. [B] SP1912 – Variant-1 and variant-2. The locations of the amino acid variations are mapped into the structures, and indicated by residue numbers and purple colouring. [C] SP2018 – The locations of the amino acid substitutions are mapped into the structure in two different colours; green for potential deleterious variations (also indicated by their residue numbers); purple for the rest.

The impact of the observed amino acid substitutions on protein structure/function and antigenicity was next assessed, to predict potential vaccine efficacy (Fig. 3, Table 2). The crystal structure of GshT (ABC transporter glutathione-binding protein) (PDB ID: 4EQ9) was used to model SP0148 variant-3. This protein exhibited 97% sequence identity to TIGR4 SP0148 between residues 31–266. Variant-3 was selected for modelling because it included 83.3% (25/30) of all the substitutions observed within the other variants. All of the substitutions identified in variant-3, in addition to 5 substitutions identified within the other SP0148 variants, were mapped to the model structure (Table 3A, Fig. 4A). A122V and N124D, identified in variant-3 only, resulted in the side chains of these residues to face inwards, whilst they faced outward in the reference protein. Furthermore, A122V and N124D led to a switch from hydrophobicity to hydrophilicity at residues 117–128 relative to TIGR4 SP0148 (Table 2, Fig. 3). Given that peak regions of hydrophilicity are hypothesised to be antigenic, variant-3 may exhibit increased antigenicity relative to TIGR4. D199A identified in SP0148 variant-4 was the only substitution predicted to be deleterious by all three SNP analysis tools (Supplementary Table S1). It is likely that pneumococci encoding this variant do not express a functional SP0148 protein. Therefore a monovalent vaccine based on SP0148 TIGR4 may not confer protection against pneumococci encoding SP0148 variant-4. Variant-4 was identified in 5% (4/76) of the Asian isolates, or <1% of the global population (4/448).

Protein NP_346341.1 from *S. pneumoniae* (PDB ID: 3M7O) with unknown function exhibited 100% sequence identity to SP1912 variant-1 (identical to SP1912 TIGR4) for residues 28 to 96 and was utilised for homology modelling. Three substitutions were identified in variant-2 relative to variant-1; R33H, E44A and V72L. R33H and V72L were not predicted to affect the protein structure (Fig. 4B). E44A was located in the helical structure of the protein; alanine has the propensity to form alpha helices and it retains the backbone structure as well as beta carbon, thus E44A may impact the interaction of variant-2 with antibodies. Variant-2 SP1912 exhibited slightly reduced hydrophilicity peaks at three regions relative to the SP1912 TIGR4, each corresponding to a three amino acid substitution, 27–35 (R33H), 38–46 (E44A) and 65–73 (V72L). SP1912 variant-2 was only identified in European isolates (15%, 11/76) representing 2% (11/445) of the isolates globally.

A maltose/maltodextrin-binding protein from *S. pneumoniae* TIGR4 (PDB ID: 2XD3), 98% identical to the SP2108 TIGR4 protein for the residues 43–423, was used to model the SP2108 variants (Fig. 4C). All SNP analysis tools agreed that D88G variant-8, D103G variant-7 and L230R were deleterious. PROVEAN and Polyphen-2 further identified D326G variant-9 as deleterious. These substitutions in variant-7, -8, -9, -12 could lead to non-functional proteins, so that a monovalent vaccine based on SP1912 TIGR4 may not confer protection against pneumococci encoding these variants. These variants were however only identified in a single study isolate (variant-7, -9 and -12 in African isolates; variant-8 in a European isolate). D326G variant-9 led to a large decrease in hydrophilicity in residues 322–330. L230R variant-12 was associated with an increase in hydrophilicity in residues 200–209. However given that these regions do not represent hydrophilic peaks in SP2108 TIGR4, it is unlikely that these allelic variants exhibit altered antigenicity to the reference sequence.

## 4 Discussion

The first requisite for a pneumococcal protein vaccine is wide distribution in the target population. We first investigated the prevalence of seven of the most promising vaccine protein candidates (PhtD, PcpA, CbpA, PspC, SP2108, SP1912 and SP0148) in the largest sequenced collection of a single serotype to date; 445 serotype 1 pneumococci isolated from four different continents, with a specific focus on isolates recovered from Africa, where serotype 1 arguably causes the greatest disease burden. PcpA, CbpA, PhtD and PspC were not present in all of the serotype 1 population. Furthermore, there was variation in the prevalence of these proteins within serotype 1 pneumococci recovered from different continents. For example, consistent with an earlier study which reported PcpA was widely distributed in French serotype 1 isolates but absent in African serotype 1 isolates [23], we report that 82% of European but only 2% of African serotype 1 pneumococci encoded PcpA. This would plausibly reduce the impact of a vaccine based on PcpA alone, as although it would provide very high coverage against serotype 1 pneumococcal disease in Europe, it would not protect against serotype 1 pneumococcal disease in Africa. Our findings highlight the need to take geographic variation into account when designing worldwide interventions strategies, as if the distribution of a protein vaccine candidate was assessed based on a dataset recovered from a single geographic region alone, it may lead to an over or under estimation of global vaccine coverage. Our results suggest a monovalent vaccine of PcpA, CbpA, PhtD and PspC would not provide universal coverage against serotype 1 pneumococci. Multi-valent protein vaccines have been advocated to increase coverage against meningococcal disease relative to vaccines based on a single protein alone [24]; we therefore investigated if a vaccine combining multiple proteins would provide improved coverage against serotype 1 pneumococci. A quad-valent formulation based on PcpA, CbpA, PhtD and PspC would provide the highest coverage against serotype 1 globally (86%), 10% higher than a mono-valent vaccine based on CbpA alone (76%). However, this would still leave a significant reservoir of serotype 1 pneumococci that could potentially evade vaccine induced immunity and which could lead to replacement disease following global protein vaccine introduction.

SP0148, SP1912 and SP2108 showed 100% distribution in the serotype 1 population, thus a vaccine based on one or more of these proteins would potentially provide 100% coverage against serotype 1 worldwide. A second requisite for an efficacious pneumococcal vaccine is minimal variation of the target antigen. SP1912 was largely conserved, with a single amino acid variant dominating globally, which showed 100% homology to the TIGR4 reference protein. In contrast, multiple variants of SP0149 and SP2108 were identified in the study population. The distribution of these variants varied between continents. SP0148 variant-1 dominated in Africa and Asia, whilst SP0148 variant-2 dominated in Europe and South America. Likewise SP2108 variant-1 was dominant in Africa and Asia, whilst SP2108 variant-3 was dominant in Europe in South America. The dominance of specific variants in the African and Asian population that are not widely distributed in isolates from either Europe or South America is consistent with whole genome analysis of a this dataset of serotype 1 pneumococci, which reported that serotype 1 forms four, genetically distinct lineages, each of which is predominantly associated with a single continent [18].

The presence of multiple variants in the target population can reduce the efficaciousness of a protein vaccine if the variants are antigenically or structurally different from the protein variant on which the vaccine is based. This can result in pneumococci encoding a specific variant escaping recognition by memory T-cells following immunisation with the vaccine variant. SP0148 variant-3, identified in only 2% of the study population was predicted to exhibit a different antigenicity profile from the *S. pneumoniae* TIGR4 reference. Furthermore, analysis at the structural level suggested that the variation identified in variant-3 might alter protein structure/function. Whilst structural analysis of SP0148 variant-4, present in 1% of the study population, suggested that isolates encoding this variant would likely express a non-functional protein. SP1912 variant-2 present in only 2% of the global population was predicted to have an altered antigenicity profile to the SP1912 TIGR4. Isolates harbouring SP2108 variants-7, -8, -9 and -12 were also predicted to express non-functional proteins. However, as each of these variants predicted to exhibit altered antigenicity/structural profiles only accounted for a very small proportion of the overall population, it is unlikely that they would have an initial impact on vaccine efficacy. Most research is now focused on a trivalent formulation of all three proteins (SP0148, SP1912 and SP2108), we therefore hypothesise that, if immunogenic, a vaccine targeted against one or more of these three proteins may illicit near universal protection against all of the variants identified within the serotype 1 population. As stated earlier however, protein vaccine candidates aim to avoid Red Queen dynamics, i.e. the opportunity for the pneumococcus to escape vaccine selective pressure by targeting universally present proteins. It is feasible that over time, the small proportion of pneumococci that already encode the non-functional or potential ‘vaccine escape’ protein variants may become more dominant in the population and ultimately reduce long term vaccine efficacy. A further potential future problem in employing these three proteins as protein vaccine candidates is that sequences with high amino acid identity to these proteins, which are hypothesised to perform the same function, have been identified in other Streptococcus species [25]. As such, it is feasible that the highly recombinogenic *S. pneumoniae* may exchange SP0148/SP1912/SP2108, for homologous proteins from other co-colonising Streptococcus species in the future and will subsequently be able to evade a vaccine based on these proteins.

Our study is limited in that the study collection was compiled as part of an African consortium and as such features predominantly African serotype 1 pneumococci and a fewer number of isolates from a limited number of countries within other continents. Thus the observations for Asia, Europe and South America serotype 1 pneumococci may be less representative of pneumococcal diversity within these regions because of limited numbers of samples/countries included and conversely the African sites may add a different bias as a consequence of contributing large numbers from a few major surveillance sites. We applied a stringent QC process to the whole genome sequences, however it is feasible that poor genome assembly in the regions encoding vaccine targets antigens, may have caused us to report that a vaccine candidate was absent in an isolate, when it was actually present. Our protein modelling and antigenicity profile analysis was based on computational predictions, furthermore the modelling was based on secondary structure analysis alone and the antigenicity profiling based on a single measure of antigenicity, thus our results may not be truly representative of protein structure and antigenicity *in vitro*. Nonetheless, this analysis highlights the fact that the proposed vaccine candidates are not universally present or conserved amongst the serotype 1 pneumococci between different geographical regions. It is therefore imperative that genomic, structural and antigenic variation between serotype 1 pneumococci between different geographical regions is taken into account when designing vaccine interventions, in order to ensure that an intervention is effective worldwide.

We report that within the serotype 1 pneumococcal population worldwide, three of the seven protein vaccine candidate antigens investigated (SP0148, SP1912, SP2108) were present in all isolates in this study, and are highly conserved at the sequence level. These candidates are currently in phase 2a clinical trials [26]; this study strongly supports their inclusion in the development of vaccines against this important pneumococcal serotype.

## Funding

This work supported by the Bill and Melinda Gates Foundation (Grant No. OPP1023440), Wellcome Trust (Award No. 084679/Z/08/Z), National Research Foundation of South Africa (Grant No. 93690) and the NIH Common Fund Award (H3A Bionet) (Grant No. U41HG006941). The content of this publication is solely the responsibility of the authors and does not necessarily represent the official views of the funders.

## Conflicts of interest

None.

## Figures and Tables

**Table 1:** *In silico* PCR primers used to identify genes encoding *S. pneumoniae* protein vaccine candidates in a global dataset of 445 serotype 1 isolates.

Target gene	Primer pair⁎	NCBI reference accession	Genomic coordinates of the gene
*cbpA*	5′-GGAAGTCAGTATTAACTAGTTATATTAGGTT	NC_011900.1	896104…898620
	3′- TGTTTATTTCCTTCTATATTTTTTCTTTAAC		
*pcpA*	5′-TCAGAATGATTAGATTTAGCTAATGGATACC	NC_011900.1	2108466…2110451
	3′-TTTAAATTTCCTTACATATTTATTTTCTAAT		
*phtD*	5′-TTCTAGCAGAAGAATTAAAGTGAGGAAAGAA	NC_011900.1	119801…121630
	3′-GTAAAATGAATGGAGCATATTTTATGGAGAA		
*pspA*	5′AAATGACTATCAGAAAAGAGGTAAATTTAGA	NC_017593.1	2170171…2172795
	3′AGCCGATTAAATTAAATCATGTTAAGAACAT		
*sp0148*	5′-GGAAGTCAGTATTAACTAGTTATATTAGGTT	NC_011900.1	2076464…2077735
	3′-TGTTTATTTCCTTCTATATTTTTTCTTTAAC		
*sp1912*	5′-ATGCTAATTCTTCTAAACTTGCTGGCTGTAT	NC_011900.1	1882330…1882620
	3′-TACCATTCATTTTAACACAAAAAAGGCTTCA		
*sp2108*	5′-AAACTTGCTATTCTTTGGGAGGAATACACTA	NC_011900.1	150062…150892
	3′-TGTTTATTTCCTTCTATATTTTTTCTTTAAC		

⁎ Derived from the 31 nucleotides flanking the 3′ and 5′ of the target gene in the reference genome.

**Table 2:** The predicted % coverage of multi-valent protein vaccine based on combinations of two or more of CbpA, PcpA, PhtD and PspA would provide against serotype 1 pneumococci by continent and also globally.

Protein combination	Asia (n = 76)	Africa (n = 324)	Europe (n = 28)	South America (n = 17)	Global (n = 445)
CbpA, PcpA	86	75	96	82	79
CbpA, PhtD	86	75	96	82	78
CbpA, PspA	84	80	68	65	79
PcpA, PhtD	29	34	82	59	37
PhtD, PspA	78	77	75	47	76
CbpA, PcpA, PhtD	84	83	96	82	84
CbpA, PcpA, PspA	84	80	96	82	82
PcpA, PhtD, PspA	80	78	96	82	80
CbpA, PcpA, PhtD, PspA	86	85	96	82	86

**Table 3:** Amino acid variations in three *S. pneumoniae* protein vaccine candidates [A] SP0148, [2] SP1912 and [C] SP2108, among a global dataset of serotype 1 pneumococci. The tables show the amino acid residues at which substitutions have been identified when using the corresponding protein sequences from *Streptococcus pneumonia*e TIGR4 as a reference. ‘.’ Indicates that a residue is identical to the TIGR4 reference sequence. Residues highlighted in grey indicate regions where the amino acid variation is predicted to result in a change to the antigenicity profile of the protein. Underlined residues indicate that the amino acid variation is predicted to be deleterious to the protein. Amino acid numbering starts at the translation site (the methionine) of the TIGR4 reference sequences.






